# Supplementary figures and images for: Co-differential genes between DKD and aging: implications for a diagnostic model of DKD
Source: PeerJ. 2024 Feb 29;12:e17046. doi: 10.7717/peerj.17046 (PMC10909364; doi:10.7717/peerj.17046)

A

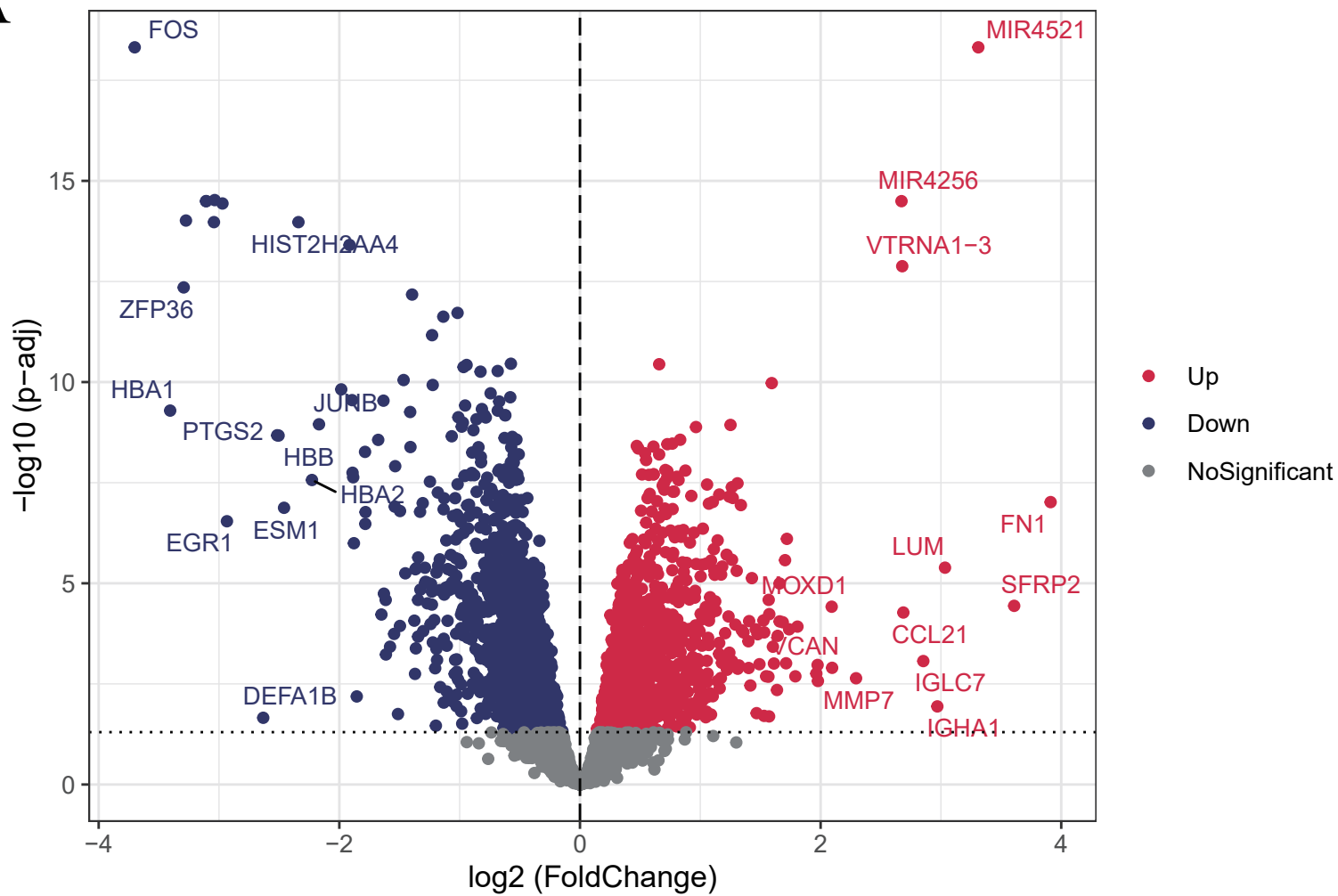

B

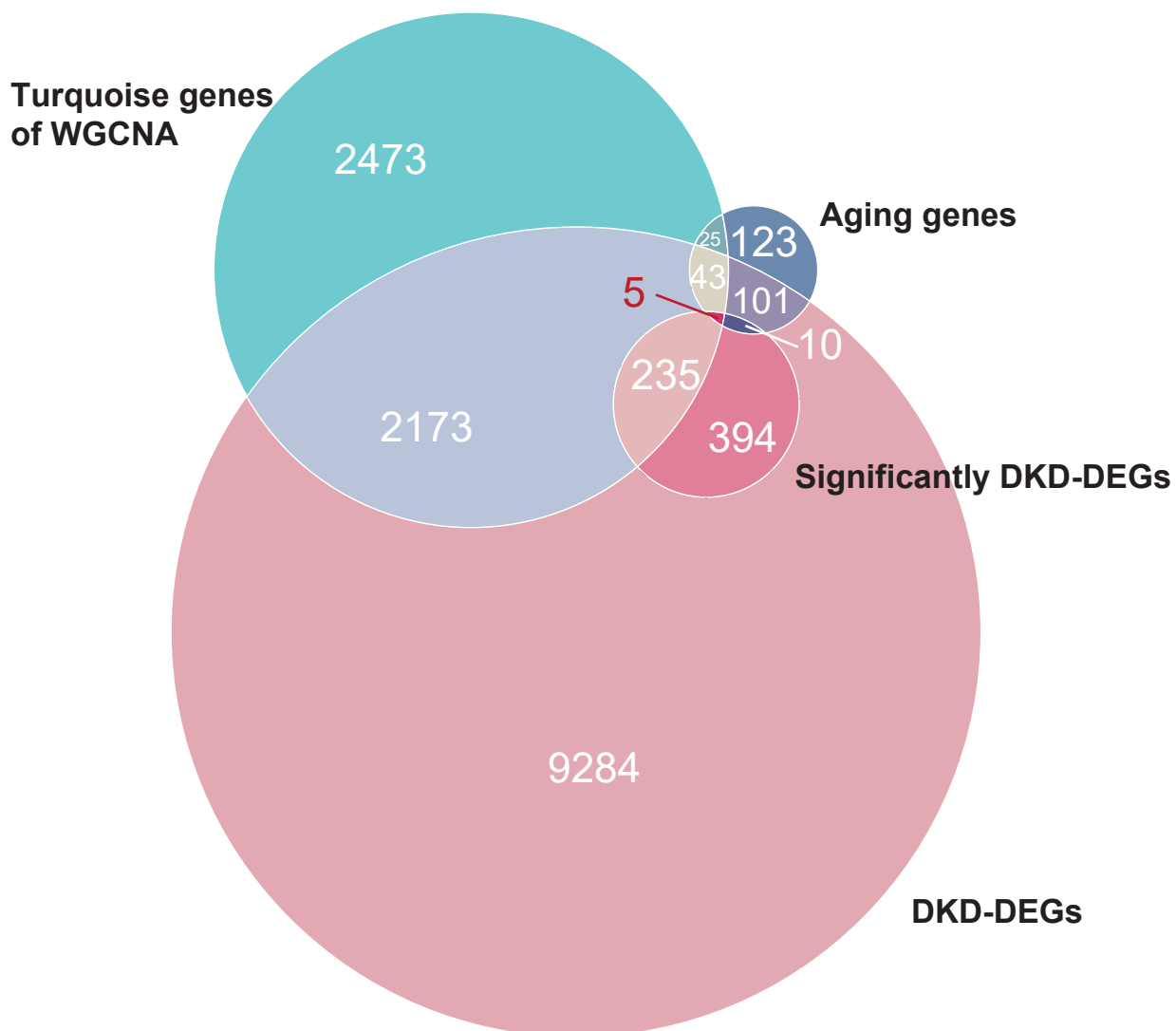

Supplement: Figure S1 — (A) Volcano plot showing the expression of turquoise module genes of DKD vs. control in GSE96804. Red and blue dots represent significantly upregulated and downregulated genes, respectively. Genes that met —LogFC— > 1 are indicated by gene names. (above the dotted line, p < 0.05). (B) Venn diagram showing 4954 turquoise module genes intersected with 12245 DKD-DEGs, 644 significant DKD-DEGs, and 307 aging-related genes. [file peerj-12-17046-s001.pdf]
