# Supplementary material for: Co-differential genes between DKD and aging: implications for a diagnostic model of DKD
Source: PeerJ. 2024 Feb 29;12:e17046. doi: 10.7717/peerj.17046 (PMC10909364; doi:10.7717/peerj.17046)
Supplement: Table S1 [file peerj-12-17046-s002.docx]

**Table S1. Statistical analysis of Relative mRNA expression value of mice kidney RT-qPCR**

| **Gene** | **Group** | **Mean value** | **T** | **Z** | **P value** | |
| --- | --- | --- | --- | --- | --- | --- |
| Igf1 | Control | 1.02923359 | 3.141 | - | 0.035 ^a^ | |
|  | DKD | 0.32522039 |  |  |  |  |
| Fos | Control | 1.13672226 | 1.606 | - | 0.184 ^a^ | |
|  | DKD | 0.49920071 |  |  |  |  |
| Pck1 | Control | 1.4031368 | 0.396 | - | 0.712 ^a^ | |
|  | DKD | 1.10611972 |  |  |  |  |
| Hspa1a | Control | 1.09936008 | - | -1.528 | 0.127 b | |
|  | DKD | 0.56833684 |  |  |  |  |
| **^a^** Significance (2-tailed) by Student’s t test. **^b^** Asymptotic significance (2-tailed) by Mann-Whitney test. | | | | | |  |
