# Supplementary material for: Co-differential genes between DKD and aging: implications for a diagnostic model of DKD
Source: PeerJ. 2024 Feb 29;12:e17046. doi: 10.7717/peerj.17046 (PMC10909364; doi:10.7717/peerj.17046)

1. **Melt Curve**
   1. Melt Peaks of β-Actin


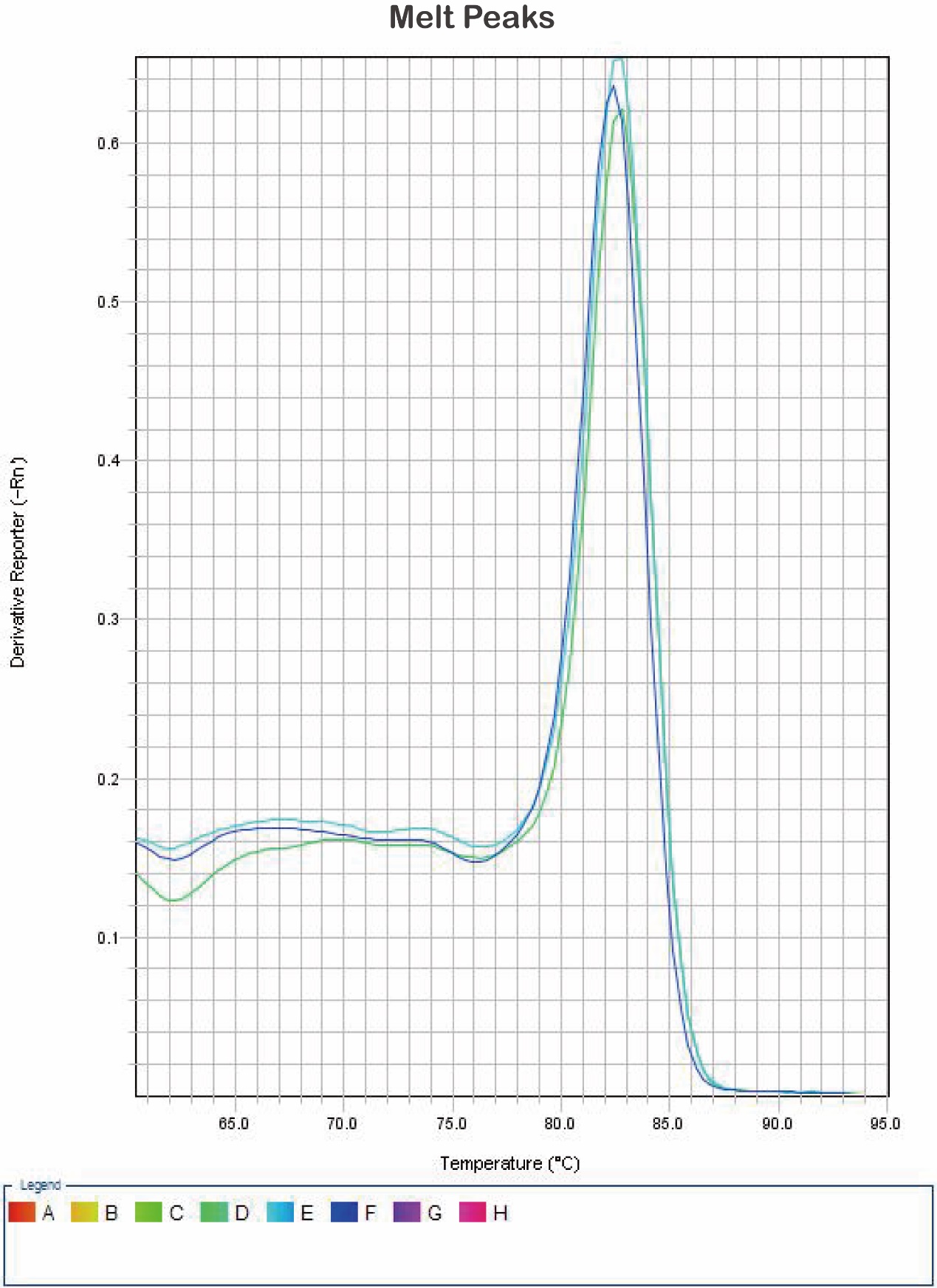


- 1. Melt Peaks of Igf1


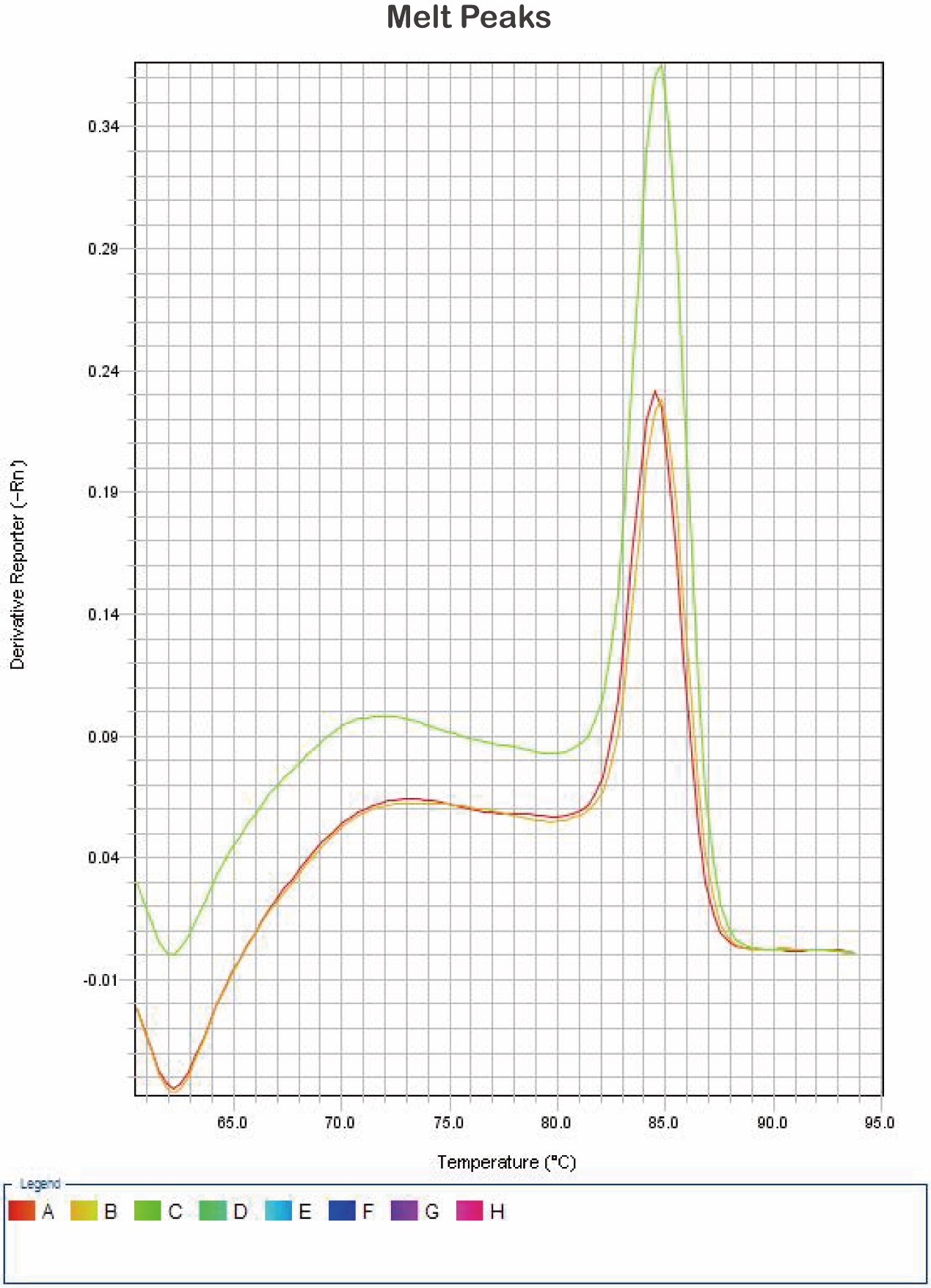


**2. Amplification Plot**

2.1 Amplification Plot of β-Actin with Threshold line


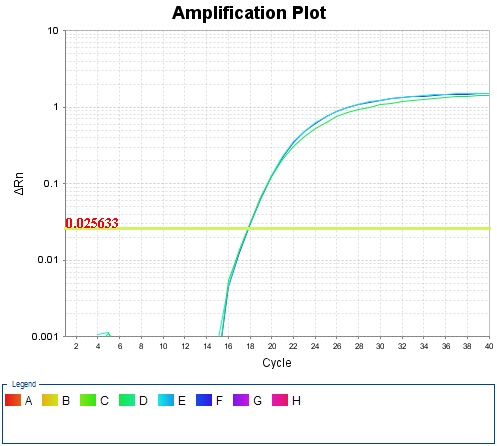


2.2 Amplification Plot of Igf1 with Threshold line


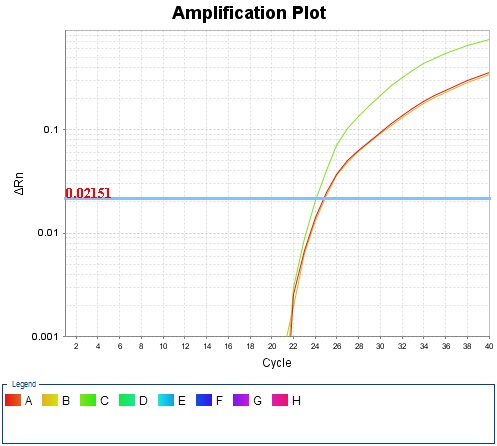


**3. Standard Curve**


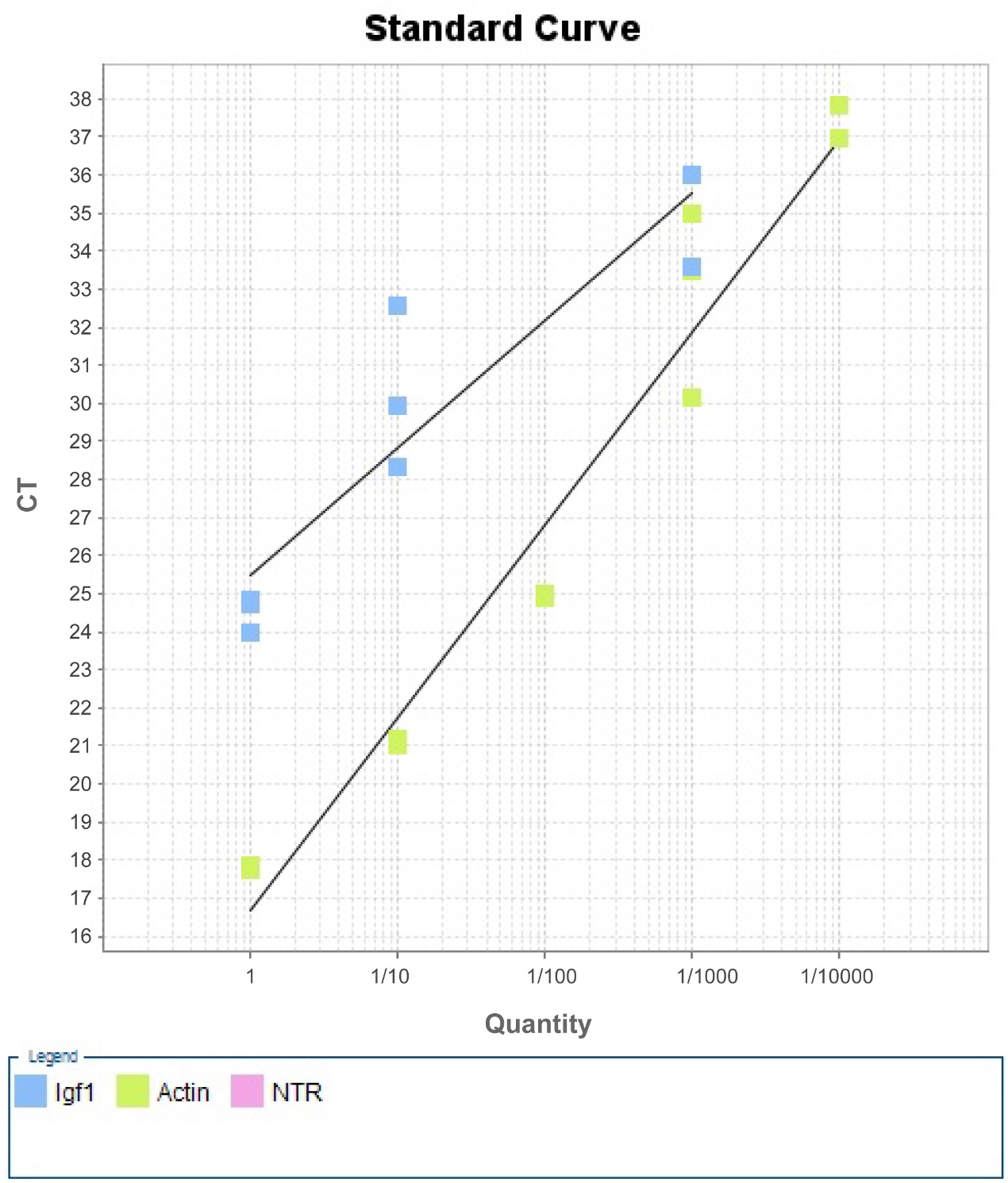


r2 of standard curve: 0.954

**4.Results of NTCs**

did not amplify.

**5. Multicomponent Plot**

5.1 Multicomponent Plot of β-Actin


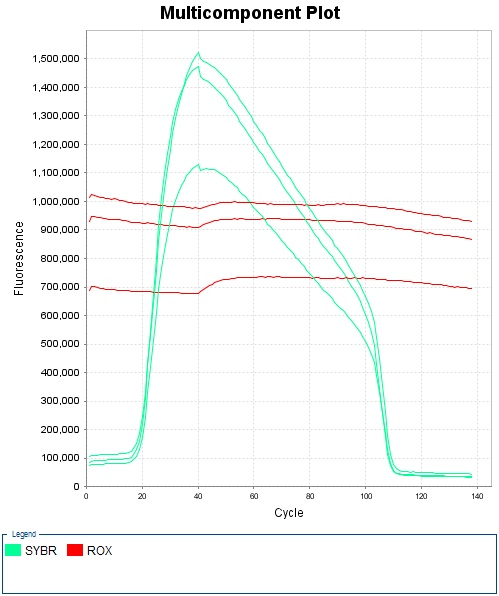


5.2 Multicomponent Plot of Igf1


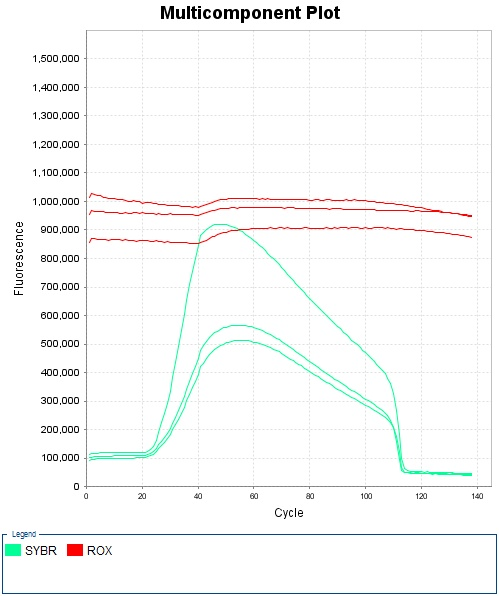

Supplement: Supplemental Information 3 [file peerj-12-17046-s003.docx]
