# Supplementary material for: Co-differential genes between DKD and aging: implications for a diagnostic model of DKD
Source: PeerJ. 2024 Feb 29;12:e17046. doi: 10.7717/peerj.17046 (PMC10909364; doi:10.7717/peerj.17046)
Supplement: Supplemental Information 4 [file peerj-12-17046-s004.pdf]

1. The RT-qPCR Raw data of mice kidney

Sample	Gene	Relative mRNA expression	Sample_Group
**con-4**	Igf1	1.346242327	Control
**con-5**	Igf1	1.136732555	Control
**con-6**	Igf1	0.604725884	Control
**DKD-10**	Igf1	0.250338054	DKD
**DKD-12**	Igf1	0.38244429	DKD
**DKD-13**	Igf1	0.342878822	DKD
**con-4**	Hspa1a	1.935573239	Control
**con-5**	Hspa1a	0.827491489	Control
**con-6**	Hspa1a	0.535015516	Control
**DKD-10**	Hspa1a	0.67886322	DKD
**DKD-12**	Hspa1a	0.505554316	DKD
**DKD-13**	Hspa1a	0.520592972	DKD
**CON4**	Fos	0.398201277	Control
**CON5**	Fos	1.56037763	Control
**CON6**	Fos	1.451587879	Control
**DKD10**	Fos	0.347063209	DKD
**DKD12**	Fos	0.367217445	DKD
**DKD13**	Fos	0.783321464	DKD
**CON4**	Pck1	2.734622384	Control
**CON5**	Pck1	1.15937589	Control
**CON6**	Pck1	0.315412115	Control
**DKD10**	Pck1	1.124233334	DKD
**DKD12**	Pck1	0.677917811	DKD
**DKD13**	Pck1	1.516208	DKD

2. Statistical analysis (Kolmogorov-Smirnov test, Homogeneity test of variance, T test and Mann-Whitney test) of the RT-qPCR data

Kolmogorov-Smirnov test

Gene	sig.
**Fos**	0.178391461
**Hspa1a**	0.03095856
**Igf1**	.200*
**Pck1**	.200*

**. This is the lower limit of true significance.*

Homogeneity test of variance

Gene	sig.
**Fos**	0.089364821
**Hspa1a**	0.042004514
**Igf1**	0.069407758
**Pck1**	0.165684596

T test

Gene	Group	Mean value	T	Sig. (2-tailed)
Igf1	Control	1.029233589	3.141	0.035
	DKD	0.325220389		
Fos	Control	1.136722262	1.606	0.184
	DKD	0.499200706		
Pck1	Control	1.403136796	0.396	0.712
	DKD	1.106119715		

Mann-Whitney test

Gene	Group	Mean Rank	Z	Asymp. Sig. (2-tailed)	Exact sig. [2*(1-tailed sig.)]
Hspa1a	Control	4.666666667	-1.52753	0.126630458	.200[b]
	DKD	2.333333333			

b. No corrected for ties.
